# Necessity to Measure PCBs and Organochlorine Pesticide Concentrations in Human Umbilical Cords for Fetal Exposure Assessment

**DOI:** 10.1289/ehp.7330

**Published:** 2004-12-14

**Authors:** Hideki Fukata, Mariko Omori, Hisao Osada, Emiko Todaka, Chisato Mori

**Affiliations:** ^1^Department of Environmental Medical Science,; ^2^Environmental Health Science Project for Future Generations,; ^3^Department of Reproductive Medicine, and; ^4^Department of Bioenvironmental Medicine, Graduate School of Medicine, Chiba University, Chiba, Japan; ^5^Department of Obstetrics and Gynecology, Chiba University Hospital, Chiba, Japan; ^6^Center for Environment, Health and Field Sciences, Chiba University, Kashiwa, Japan

**Keywords:** cord blood, maternal blood, organochlorine pesticides, polychlorinated biphenyls (PCBs), umbilical cord

## Abstract

Three types of tissue samples—umbilical cord (UC), umbilical cord serum (CS), and maternal serum (MS)—have often been used to assess fetal exposure to chemicals. In order to know the relationship of contamination between mothers and fetuses, we measured persistent chemicals in comparable sets of the three tissue samples. Also, we analyzed the association between the chemicals in maternal and fetal tissues to know which tissue is the best sample for fetal exposure assessment. On a wet basis, the chemical concentrations were of the order MS > CS > UC, except for some chemicals such as *cis*-chlordane and endosulfan. On a lipid basis, the concentrations in UC were nearly equal or often higher than in MS, but the concentrations in CS were usually lower than in others. Hexachlorocyclohexanes and penta-, hexa-, and heptachlorinated biphenyls showed an association between the concentrations in UC versus MS, and UC versus CS. These chemicals also showed high correlation coefficients between the chemical concentrations in UC of first babies and maternal age. These chemicals were closely related to each other when grouped on the basis of their concentrations using cluster analysis. In conclusion, we insist that UC is the best sample to assess fetal contamination status of persistent chemicals. There is a possibility that the assessment based on the contamination levels in CS result in an underestimation.

It is believed that humans are exposed to multiple chemicals from food, air, water, and so forth, including natural products; industrial products, such as polychlorinated biphenyls (PCBs), pesticides, and pharmaceuticals; and nonintentional products, such as dioxins. Human fetuses are exposed to multiple chemicals through placenta in Japan (Mori 2001; Mori et al. 2003; Todaka and Mori 2002), and infants are exposed to these chemicals through milk (Borgert et al. 2003). A number of persistent organochlorine pollutants have been detected in human follicular fluid (De Felip et al. 2004) and amniotic fluid (Foster et al. 2000). Because human fetuses and infants are considered significantly more sensitive to a variety of environmental toxicants compared with adults (Branum et al. 2003; Charnley and Putzrath 2001; Needham and Sexton 2000), the adverse effects of chemicals on these fetuses and infants are of concern.

Three types of tissue samples—umbilical cord (UC), umbilical cord serum (CS), and maternal serum (MS)—have often been used to assess fetal exposure to chemicals. There are several reports indicating that the chemical concentrations were higher in maternal blood than in cord blood (Sarcinelli et al. 2003; Waliszewski et al. 2000; Walker et al. 2003). The assessments using cord blood have suggested fetal contamination. However, the chemical concentrations in fetal tissues are still unclear. There are only a few reports using fetal tissues such as UC (Covaci et al. 2002; Grandjean et al. 2001).

In order to know the relationship of contamination between mothers and fetuses, we measured persistent chemicals in comparable sets of the three tissue samples (UC, CS, and MS). Also, we analyzed the association between the chemicals in maternal and fetal tissues to know which tissue is the best sample for fetal exposure assessment.

## Materials and Methods

### Sample.

Thirty-two pregnant women who were general citizens and lived in the cities of Chiba and Yamanashi, near Tokyo, Japan, were surveyed in 2002 and 2003. UC (~ 20 cm), maternal blood (10 mL), and cord blood (10 mL) were collected from the cases delivered by cesarean section. The deliveries were conducted at least 12 hr after the last meal. UC without cord blood and MS and CS were stored at −20°C until use in glassware that had been checked to be without contamination. In the whole-study subjects, 20 mothers had complete samples (MS, CS, and UC), and their average age at delivery was 32.8 ± 4.0 years. There were 12 mothers without CS samples, and their average age was 31.9 ± 4.9 years. In total, 32 cases were used for the analysis of correlation between maternal age and chemical concentrations. This study has been approved by the Congress of Medical Bioethics of Chiba University and the University of Yamanashi, and all the samples were obtained after receipt of written informed consent.

### Chemicals measured.

We measured 19 organochlorine pesticides: dichlorodiphenyl-trichloroethane (DDT) and its metabolites [dichlorodiphenyldichloroethylene (DDE) and dichlorodiphenyldichloroethane (DDD): *p*,*p*′-DDT, *o*,*p*′-DDT, *p*,*p*′-DDE, *o*,*p*′-DDE, *p*,*p*′-DDD, *o*,*p*′-DDD], chlordane and its metabolites (*cis*-chlordane, *trans*-chlordane, *trans*-nonachlor, oxychlordane), heptachlor and its metabolites (heptachlor, heptachlor epoxide), methoxychlor, “drins” (dieldrin, aldrin, endrin), endosulfan isomers (mixture of α- and β-endosulfan), hexachlorobenzene (HCB), and hexachlorocyclohexane (HCH) isomers (mixture of α-, β-, γ-, and δ -HCH). We also measured 10 groups of PCB congeners grouped by their number of chlorines from 1 to 10.

### Pretreatment.

MS (4–5 mL), CS (3–4 mL), and UC (17–27 g) were used for the preparation of samples for gas chromatography–mass spectrometry. The details were revealed to the public through the homepage of the Ministry of Environment of the Government of Japan (2002). Briefly, the UC samples were homogenized with ethanol/hexane (1:3) and sodium sulfuric anhydride by a Polytron PT3100 (Kinematica AG, Littau-Lucerne, Switzerland) after ^13^C_12_-labeled PCB, ^13^C_6_-labeled β-HCH, ^13^C_6_-labeled HCB, ^13^C_9_-labeled endosulfan-I, ^13^C_12_-labeled pentaCB, and ^13^C_12_-labeled *p*,*p*′-DDT had been added as quantitative standards. After filtration, the filtrate and an additional filtrate of the rehomogenate of the residue were washed with water twice. The resulting hexane extract was dehydrated using sodium sulfuric anhydride and concentrated by evaporation. One-sixth of the concentrated extract was used for measurement of PCBs, another sixth for that of organochlorine pesticides, and half for the gravimetric fat determination.

The MS and CS samples were extracted twice using an ether/hexane (3:1) mixture after addition of the quantitative standards. The resulting ether/hexane extract was dehydrated using sodium sulfuric anhydride and concentrated by evaporation (crude extract). A fourth the crude extract was used for measurement of PCBs, and another fourth for organochlorine pesticides.

### Measurement of PCBs.

After the crude extract was treated with 1 mol/L KOH/ethanol for 18 hr, it was extracted using hexane three times and was concentrated with nitrogen. The concentrate was then eluted through a silica gel 60 column (70-230 ASTM-mesh; Merck, Darmstadt, Germany) with 10 mL hexane, evaporated to a final volume of 0.1 mL, and analyzed after the addition of ^13^C_12_-labeled PCB.

PCBs were quantitated by gas chromatography–mass spectrometry. Gas chromatography was performed using a Hewlett Packard HP6800 series equipped with a Micromass AutoSpec Ultima mass spectrometer (Micromass Ltd., Manchester UK). An HT8 fused silica capillary column [0.25 mm inner diameter (i.d.) × 25 m with a 0.33-mm film thickness; SGE International Pty Ltd., Austin, TX, USA] was used to separate each PCB congener. The column temperature was maintained at 100°C for 2 min, raised to 180°C at a rate of 5°C/min, maintained at 180°C for 0.5 min, raised to 270°C at a rate of 20°C/min, then to 300°C at a rate of 5°C/min, and finally maintained at 300°C for 2 min. The carrier gas (helium) flow rate was 1 mL/min. The ionizing current was 600 μA, the ionizing energy was 38 eV, and the accelerating voltage was 8 kV. The resolution of the mass spectrometer was maintained at approximately > 10,000 (10% valley) throughout, and the analysis was carried out according to selected ion monitoring.

### Measurement of organochlorine pesticides.

The crude extract was evaporated to a final volume of 0.5 mL and extracted twice with hexane-saturated acetonitrile. The resulting acetonitrile extract was added to water and extracted with hexane twice, then dehydrated using sodium sulfuric anhydride, and evaporated with nitrogen. The concentrate was eluted through a Florisil column (1 g/6 cc, Seppak Vac Florisil; Waters, Milford, MA, USA) using 10 mL hexane, evaporated to a final volume of 0.1 mL, and analyzed after the addition of fluoranthene-d10.

Organochlorine pesticides were quantitated by gas chromatography-mass spectrometry in the same manner as for PCBs, except that the column was a BPX-25 fused silica capillary column, 0.22 mm i.d. × 30 m with a 0.25-mm film thickness (SGE International Pty Ltd.). The column temperature was maintained at 60°C for 1 min, raised to 300°C at a rate of 10°C/min, and finally maintained at 300°C for 10 min.

### Lipid contents.

Lipid contents in the UC samples were determined gravimetrically, and lipid contents in MS and CS were determined enzymatically as the sum of the total cholesterol, triglycerides, and phospholipids.

### Statistical analysis.

The statistical analysis was performed using Microsoft Excel 2002 (Microsoft, Redmond, WA, USA). Cluster analysis was performed by the cosine correlation method using GeneMaths software (version 1.50; Applied Maths BVBA, Sint-Martens-Latem, Belgium).

## Results

### Detection rate.

Tables 1–4 show the concentration of organochlorine pesticides (Tables 1 and 2) and PCBs (Tables 3 and 4) in the three types of tissues (MS, CS, and UC). It became clear that human fetuses were contaminated with multiple chemicals in Japan. However, *o*,*p*′-DDE, *o*,*p*′-DDD, aldrin, endrin, and methoxychlor were not detected in any of the tissues in this study. Other chemicals were detected in MS and/or UC, but the detection rate was very low in the CS (Tables 1 and 2). In particular, *cis*-chlordane, endosulfan, *p*,*p*′-DDT, dieldrin, *p*,*p*′-DDD, and heptachlor were not detected in CS; however, both were detected in MS (detection rate > 70%) and UC. PCB congeners with five to seven chlorines were detected in all samples, whereas other congeners showed a relatively low detection rate in CS (Tables 3 and 4).

### Contamination levels.

The highest concentrations found were *p*,*p*′-DDE, HCHs, and HCB in all three tissues both on a lipid basis (Table 1) and wet basis (Table 2). Generally, the chemical concentrations on a wet basis were of the order MS > CS > UC. This is due to the difference in lipid content. Lipid content in MS, CS, and UC (20 complete samples) was 0.76 ± 0.13%, 0.23 ± 0.04%, and 0.11 ± 0.02%, respectively. Remarkably, the concentrations of some chemicals in UC on a wet basis, such as *cis*-chlordane and endosulfan, were almost equal to those in MS (Table 2). On the other hand, on a lipid basis, the concentrations of the following chemicals in UC were nearly equal or often higher than in MS: HCHs, *p*,*p*′-DDT, *cis*-chlordane, *trans*-chlordane, endosulfan, and heptachlor epoxide (*p* < 0.001, paired *t*-test; Table 1). The chemical concentrations in CS were usually lower than in other tissues.

PCB congeners grouped on the basis of their number of chlorines showed different patterns of distribution depending on the number of chlorines. TetraCB and pentaCB concentrations were higher in MS than in UC (*p* < 0.05, paired *t*-test), whereas hexaCB and heptaCB concentrations were higher in UC than in MS (*p* < 0.001, paired *t*-test) on a lipid basis (Table 3); particularly, heptaCBs and octaCBs showed a high UC:MS ratio. The UC:MS ratio varied according to the number of chlorines in the range of 3–8: UC < MS for congeners with 3–5 chlorines, and UC > MS for congeners with 6–8 chlorines (Table 3). The detection rates of congeners with 1 or 2 chlorines were higher in UC than in MS, whereas those with of 9 or 10 chlorines were higher in MS than in UC. The concentrations (average and median, on a lipid basis) of congeners with 9 or 10 chlorines were higher than those with 1 or 2 chlorines in MS, whereas concentrations of congeners with 1 or 2 chlorines were higher than those with 9 or 10 chlorines in UC. These facts suggest that the accumulation of PCBs in UC is different depending on the number of chlorines; PCB congeners with 1, 2, 6, 7 and 8 chlorines easily accumulate in UC compared with other congeners.

### Association between the chemical concentrations among chemicals.

**Figure 1:**
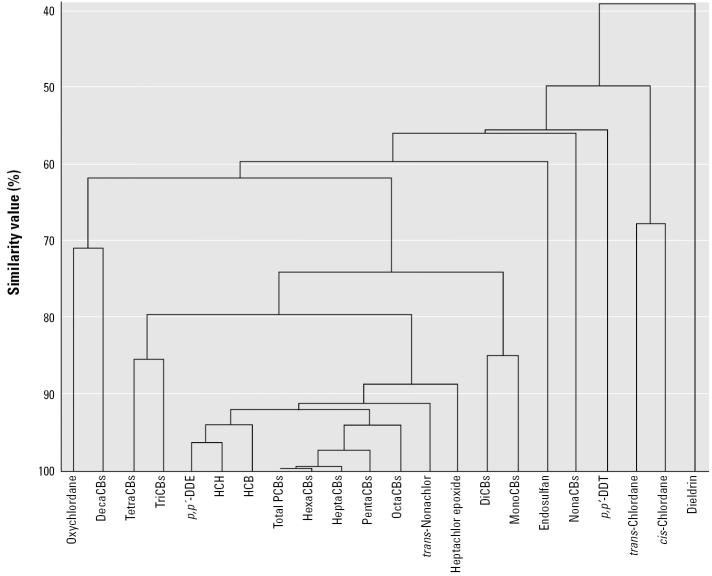
Cluster analysis of chemical concentrations among UC samples. Similarity values were calculated by cosine correlation matrix for chemical concentrations in UC for UC; the results are shown as a dendrogram.

We reported that correlation existed between total PCBs and other persistent chemicals, such as *p*,*p*′-DDE, HCB, and HCHs, in human UC (Mori et al. 2003). To confirm our previous findings, we applied a cluster analysis technique in the present study. We used the cluster analysis to discover “natural” groupings of objects that reflect evolutionary or functional relationships among the objects; some of the cluster analyses often done in toxicogenomics research have this objective (Immermann and Huang 2003). The cluster analysis was performed using cosine correlation matrix for chemical concentrations in UC for UC, and the clustering results were represented in the dendrogram (Figure 1). Consequently, we found that PCBs with 5–8 chlorines and some organochlorines, such as *p*,*p*′-DDE, HCB, and HCHs, were closely related to each other (Figure 1).

### Association between the chemical concentrations among the three types of tissues.

Correlation of organochlorine pesticide concentrations among the three types of tissues is shown in Table 5. Some organochlorine pesticides showed no association between MS versus CS and/or between CS versus UC. Between MS versus CS, HCB, HCHs, heptachlor epoxide, and PCBs with chlorines showed a relatively high correlation coefficient (*r* > 0.7). Between CS versus UC, HCHs, *p*,*p*′-DDE, and PCBs with 5–8 chlorines showed relatively high correlation (*r* > 0.7). Comparing MS and UC, HCHs and PCBs with 4–7 chlorines showed a relatively high correlation coefficient (*r* > 0.7).

### Association between the chemical concentrations in UC and maternal age.

Several studies have reported that chemical concentrations were dependent upon maternal age at delivery (Mori et al. 2003; Rhainds et al. 1999). Our present results confirmed that HCHs, pentaCBs, hexaCBs, heptaCBs, and octaCBs showed such correlation (Table 5). A significant correlation was found between CS versus UC and age versus UC (*r* = 0.75; Table 5). Also, relatively significant correlation was found between MS versus UC and age versus UC (*r* = 0.64; Table 5). However, we found no correlation between MS versus CS and age versus UC (*r* = 0.04; Table 5). That is, HCHs, pentaCBs, hexaCBs, and heptaCBs tended to show relatively high association of concentrations in CS versus UC and MS versus UC. Also, these chemicals showed high correlation coefficients between the chemical concentrations in UC of first babies and maternal age.

## Discussion

We investigated the distribution of organo-chlorine pesticides and PCBs in three types of tissues (UC, CS, and MS). We analyzed the chemical contamination status mainly on a lipid basis because the liposolubility rate is thought to be a major factor influenced by rates of accumulation and elimination from tissues and organs (Parham et al. 1997) and because the existing differences depend principally on lipid content of the tissues (Henriksen et al. 1998). Several studies have reported that the concentration levels of persistent chemicals showed association between cord blood and maternal blood (Sala et al. 2001; Waliszewski et al. 2000; Walker et al. 2003). In our study, we found strong correlation between MS versus CS (Table 5) in some organochlorine pesticides and PCB congeners. Also, Grandjean et al. (2001) showed high associations between cord blood and UC. The tendency was confirmed in our study of HCHs, *p*,*p*′-DDE, and some PCB congeners (Table 5). However, we found no report that compared the concentration levels among UC, CS, and MS. Hence, we compared the data among these three tissues.

In the present study, we found that the chemical concentrations were often higher in UC than in CS on a lipid basis, and the detection rates and the concentrations in CS were often lower than in MS and UC. In past studies, chemical concentrations were higher in adipose tissues than in serum (López-Carrillo et al, 1999; Pauwels et al. 2000), and in other studies, concentrations were higher in serum lipid than in breast tissues (Waliszewski et al. 2003). Moreover, as suggested by Pauwels et al. (2000), the concentration levels of persistent chemicals varied dramatically depending on the tissues (Tables 1 and 3). One of the reasons for the confusion may be the pharmaco-kinetics of chemicals in blood. Mohammed et al. (1990) and Norén et al. (1999) reported that chemicals in blood are bound to lipoproteins and albumin rather than being dissolved in lipid, and the distribution in plasma vary according to the chemicals. It is possible that a free form of chemicals is distributed by simple equilibrium, but distribution or transport of bound form of chemicals to protein in blood is more complicated, so the chemical concentration in CS might be lower than in MS and UC. Further studies on the distribution of contaminants in different body tissues and fetal tissues are required.

In conclusion, we believe that UC is the best sample to assess fetal contamination status of persistent chemicals. There is a possibility that assessment based on the contamination levels in CS result in an underestimation.

## Figures and Tables

**Table 1:** Organochlorine pesticide concentrations (pg/g-lipid) in three types of tissue.

Organochlorine pesticide, tissue	Detection*^a^* (%)	Mean ± SD	Minimum	25th percentile	Median	75th percentile	Maximum
HCB							
MS	100	15,500 ± 6,220	3,600	10,000	16,000	17,800	31,000
CS	100	10,900 ± 3,680**	5,200	8,800	11,000	12,000	18,000
UC	95	17,700 ± 6,360*^##^*	ND	16,000	18,000	20,000	28,000
HCHs							
MS	100	27,400 ± 10,630	13,000	22,000	26,000	30,000	55,000
CS	100	33,800 ± 19,300	12,000	24,000	28,000	39,000	100,000
UC	100	36,200 ± 14,920**	18,000	26,000	30,000	45,000	69,000
*p,p*′-DDT							
MS	80	3,380 ± 3,240	ND	1,000	2,400	5,100	11,000
CS	0	—		—	—	—	—
UC	50	5,550 ± 7,160**	ND	ND	1,100	9,300	19,000
*o,p*′-DDT							
MS	15	38 ± 104	ND	ND	ND	ND	340
CS	0	—	—	—	—	—	—
UC	0	—	—	—	—	—	—
*p,p*′-DDE							
MS	100	89,700 ± 33,600	19,000	71,000	93,000	110,000	150,000
CS	100	33,000 ± 16,500**	14,000	22,000	28,000	42,000	75,000
UC	100	79,600 ± 26,200*^##^*	29,000	64,000	78,000	90,000	140,000
*p,p*′-DDD							
MS	85	766 ± 982	ND	200	360	950	3,800
CS	0	—		—	—	—	—
UC	15	215 ± 527	ND	ND	ND	ND	1,600
*cis*-Chlordane							
MS	100	240 ± 165	63	110	200	330	660
CS	0	—		—	—	—	—
UC	70	1,220 ± 1,230**	ND	ND	1,200	1,700	4,400
*trans*-Chlordane							
MS	95	320 ± 257	ND	160	240	430	1,200
CS	20	198 ± 449	ND	ND	ND	ND	1,400
UC	55	644 ± 848**	ND	ND	290	1,200	3,000
Oxychlordane							
MS	85	2,640 ± 4,160	ND	620	1,200	3,900	19,000
CS	40	978 ± 1,630	ND	ND	ND	1,600	6,300
UC	60	2,120 ± 2,100	ND	ND	1,900	3,700	6,100
*trans*-Nonachlor							
MS	100	7,230 ± 2,840	2,000	5,600	7,000	9,000	14,000
CS	80	3,780 ± 6,470	ND	1,400	1,900	3,800	30,000
UC	100	7,660 ± 2,580	2,500	6,200	6,700	8,300	14,000
Dieldrin							
MS	70	495 ± 454	ND	ND	440	770	1,600
CS	0	—	—	—	—	—	—
UC	45	1,970 ± 3,170*	ND	ND	ND	2,200	9,600
Endosulfan							
MS	90	380 ± 267	ND	280	340	460	1,100
CS	0	—	—	—	—	—	—
UC	70	2,090 ± 2,440**	ND	ND	1,600	2,900	9,400
Heptachlor							
MS	25	150 ± 272	ND	ND	ND	100	700
CS	0	—		—	—	—	—
UC	10	505 ± 1,620	ND	ND	ND	ND	6,500
Heptachlor epoxide							
MS	100	142 ± 729	310	950	1,200	1,700	3,000
CS	95	1,580 ± 844	ND	1,100	1,500	1,900	3,400
UC	100	2,790 ± 1,280**,*^##^*	160	2,000	2,700	3,500	6,000

ND, not detected.

a Detection rate is shown as a percentage (*n* = 20).

* *p* < 0.05 and

** *p* < 0.001 compared with MS.

## *p* < 0.001 compared with CS.

**Table 2:** Organochlorine pesticide concentrations (pg/g-wet) in three types of tissue.

Organochlorine pesticide, tissue	Detection*^a^* (%)	Mean ± SD	Minimum	25th percentile	Median	75th percentile	Maximum
HCB							
MS	100	120 ± 55.2	20	81	120	140	230
CS	100	23.9 ± 8.42**	13	20	23	25	46
UC	95	19.6 ± 6.52**	ND	17	20	23	33
HCHs							
MS	100	208 ± 84.7	100	160	190	240	430
CS	100	74.5 ± 39.3**	33	49	70	82	190
UC	100	39.8 ± 7.8**^,^*^##^*	17	29	35	48	95
*pp*′-DDT							
MS	80	26.40 ± 26.5	ND	8.0	17	42	90
CS	0	—	—	—	—	—	—
UC	50	5.76 ± 7.53*	ND	ND	1.0	11	27
*op*′-DDT							
MS	15	0.32 ± 0.89	ND	ND	ND	ND	3
CS	0	—	—	—	—	—	—
UC	0	—	—	—	—	—	—
*pp*′-DDE							
MS	100	680.0 ± 277	190	480	640	900	1,200
CS	100	71.9 ± 30.7**	26	50	72	95	130
UC	100	86.5 ± 26.1**^,^*^#^*	31	76	88	94	150
*pp*′-DDD							
MS	85	6.11 ± 7.99	ND	1.6	2.7	7.1	30
CS	0	—	—	—	—	—	—
UC	15	0.26 ± 0.63	ND	ND	ND	ND	2.1
*cis*-Chlordane							
MS	100	1.81 ± 1.25	0.54	0.70	1.3	2.6	4.8
CS	0	—	—	—	—	—	—
UC	70	1.20 ± 1.19	ND	ND	1.1	2.0	4.4
*trans*-Chlordane							
MS	95	2.46 ± 2.45	ND	1.2	1.9	3.1	12
CS	20	0.41 ± 0.90	ND	ND	ND	ND	2.9
UC	55	0.68 ± 0.91*	ND	ND	0.25	1.1	2.9
Oxychlordane							
MS	85	22.80 ± 45.4	ND	4.9	11	24	210
CS	40	2.19 ± 3.45	ND	ND	ND	3.6	12
UC	60	2.48 ± 2.50	ND	ND	2.3	4.5	6.9
*trans*-Nonachlor							
MS	100	55.00 ± 23.2	15	38	53	68	100
CS	80	8.02 ± 12.1**	ND	3.1	4.3	9.7	56
UC	100	8.52 ± 3.17**	2.9	6.8	7.9	10.3	17
Dieldrin							
MS	70	3.75 ± 3.30	ND	ND	3.4	5.9	10
CS	0	—	—	—	—	—	—
UC	45	2.02 ± 3.01	ND	ND	2.7	2.6	8.5
Endosulfan							
MS	90	2.90 ± 2.07	ND	2	2.6	3.9	8.4
CS	0	—	—	—	—	—	—
UC	70	2.83 ± 2.61	ND	ND	1.9	3.3	10
Heptachlor							
MS	25	1.44 ± 2.58	ND	ND	ND	1.2	7.2
CS	0	—	—	—	—	—	—
UC	10	0.57 ± 1.82	ND	ND	ND	ND	7.2
Heptachlor epoxide							
MS	100	10.70 ± 5.67	2.3	6.9	9	13	24
CS	95	3.51 ± 1.80**	ND	2.4	3.3	4.2	7.3
UC	100	2.89 ± 1.03**	0.3	2.2	3.1	3.5	5.1

ND, not detected.

a Detection rate is shown as a percentage (*n* = 20).

* *p* < 0.05 and

** *p* < 0.001 compared with MS.

# *p* < 0.05 and

## *p* < 0.001 compared with CS.

**Table 3:** PCB concentrations (pg/g-lipid) in three types of tissue.

PCB, tissue	Detection*^a^* (%)	Mean ± SD	Minimum	25th percentile	Median	75th percentile	Maximum
MonoCBs							
MS	25	8.4 ± 30.5	ND	ND	ND	28	110
CS	0	—	—	—	—	—	—
UC	95	480 ± 405*	ND	100	470	640	1,400
DiCBs							
MS	70	44 ± 37	ND	ND	44	67	120
CS	0	—	—	—	—	—	—
UC	80	354 ± 276**	ND	220	370	450	1,200
TriCBs							
MS	100	1,630 ± 639	720	1200	1400	2,000	2,900
CS	65	1,630 ± 1770	ND	ND	1,500	2,200	6,700
UC	90	1,210 ± 977	ND	560	1100	1,800	3,900
TetraCBs							
MS	100	7,000 ± 2120	4,000	7,400	7,100	8,000	12,000
CS	95	4,360 ± 3750**	ND	1,800	3,300	5,600	14,000
UC	95	3,190 ± 2,200**	ND	2,100	3,000	4,000	10,000
PentaCBs							
MS	100	15,000 ± 4,630	7,700	11,000	15,000	18,000	25,000
CS	100	12,700 ± 6,080**	3,700	8,000	13,000	17,000	24,000
UC	100	13,200 ± 6,100*	5,100	7,900	12,000	18,000	25,000
HexaCBs							
MS	100	25,600 ± 8,410	11,000	20,000	26,000	30,000	42,000
CS	100	31,200 ± 11,000**	14,000	23,000	31,000	38,000	51,000
UC	100	32,600 ± 12,000**	13,000	24,000	35,000	40,000	53,000
HeptaCBs							
MS	100	9,640 ± 3,610	3,900	7,400	8,600	12,000	17,000
CS	100	12,300 ± 5,420**	3,500	8,000	12,000	13,500	23,000
UC	100	15,200 ± 5,860**^,^*^##^*	7,700	11,000	14,000	20,000	29,000
OctaCBs							
MS	100	1,750 ± 718	590	1,500	1,800	2,500	3,100
CS	75	1,700 ± 1,380	ND	830	1,400	2,600	4,400
UC	100	3,130 ± 1,360**^,^*^##^*	1,700	2,100	2,700	3,900	5,900
NonaCBs							
MS	70	212 ± 174	ND	ND	220	290	530
CS	35	146 ± 291	ND	ND	ND	200	1,200
UC	50	148 ± 221	ND	ND	60	210	910
DecaCBs							
MS	85	114 ± 65	ND	91	115	150	240
CS	35	130 ± 257	ND	ND	ND	220	1,100
UC	55	148 ± 173*	ND	ND	96	255	570
Total PCBs							
MS	100	61,500 ± 18,400	29,000	46,000	61,000	72,000	96,000
CS	100	63,800 ± 23,300	31,000	44,000	63,000	77,000	110,000
UC	100	70,000 ± 26,100*^,^*^#^*	34,000	47,000	73,000	88,000	130,000

ND, not detected.

a Detection rate is shown as a percentage (*n* = 20).

* *p* < 0.05 and

** *p* < 0.001 compared with MS.

# *p* < 0.05 and

## *p* < 0.001 compared with CS.

**Table 4:** PCB concentrations*^a^* (pg/g-wet) in three types of tissue.

PCB, tissue	Detection*^a^* (%)	Mean ± SD	Minimum	25th percentile	Median	75th percentile	Maximum
MonoCBs							
MS	25	0.10 ± 0.21	ND	ND	ND	0.02	0.68
CS	0	—	—	—	—	—	—
UC	95	0.52 ± 0.47	ND	0.16	0.36	0.82	1.7
DiCBs							
MS	70	8.65 ± 0.28	ND	ND	0.35	0.52	0.89
CS	0	—	—	—	—	—	—
UC	80	0.38 ± 0.30	ND	0.24	0.36	0.50	1.2
TriCBs							
MS	100	13.0 ± 6.14	6.1	8.5	10	15	31
CS	65	3.78 ± 4.42**	ND	ND	3.6	4.8	17
UC	90	1.38 ± 1.15**^,^*^#^*	ND	0.45	1.3	1.8	4.4
TetraCBs							
MS	100	53.2 ± 17.75	22	40	50	65	91
CS	95	9.59 ± 8.32**	ND	4.2	6.9	13	35
UC	95	3.69 ± 3.13**^,^*^##^*	ND	2.4	2.9	4.5	15
PentaCBs							
MS	100	115 ± 42.3	51	81	110	140	190
CS	100	27.5 ± 12.8**	8.6	19	26	34	54
UC	100	14.6 ± 7.54**^,^*^##^*	5.2	10	13	18	37
HexaCBs							
MS	100	195 ± 68.4	90	140	210	240	340
CS	100	69.3 ± 25.9**	26	51	68	88	130
UC	100	36.1 ± 15.3**^,^*^##^*	16	26	35	43	78
HeptaCBs							
MS	100	73.3 ± 26.7	34	55	73	94	120
CS	100	27.9 ± 13.8**	6.2	19	26	32	61
UC	100	17.1 ± 8.26**^,^*^##^*	8.6	12	16	20	43
OctaCBs							
MS	100	14.5 ± 5.57	4.6	11	15	18	26
CS	75	4.03 ± 3.96**	ND	1.4	3.4	5.1	16
UC	100	3.57 ± 1.86**^,^*^#^*	1.4	2.1	3.4	4.3	8.3
NonaCBs							
MS	70	1.54 ± 1.26	ND	ND	1.6	2.5	3.8
CS	35	0.32 ± 0.64	ND	ND	ND	0.52	2.7
UC	50	0.17* ± 0.27*	ND	ND	0.065	0.26	1.1
DecaCBs							
MS	85	0.87 ± 0.50	ND	0.73	0.86	1.0	1.7
CS	35	0.29 ± 0.58	ND	ND	ND	0.49	2.5
UC	55	0.18 ± 0.23**	ND	ND	0.094	0.33	0.85
Total PCBs							
MS	100	467 ± 154	220	340	490	570	780
CS	100	139 ± 56.4**	56	100	130	180	270
UC	100	77.4 ± 35.5**^,^*^##^*	35	57	73	91	190

ND, not detected.

a Detection rate is shown as a percentage (*n* = 20).

* *p* < 0.05 and

** *p* < 0.001 compared with MS.

# *p* < 0.05 and

## *p* < 0.001 compared with CS.

**Table 5:** Correlation coefficients (*r*) of chemicals among tissues and between maternal age and chemical concentrations in UC of first babies.

	Among tissues*^a^*			Between maternal age and concentrations in UC of first babies (age vs. UC)
	MS vs. CS	CS vs. UC	MS vs. UC		
HCB	0.73	0.30	0.39	−0.16
HCHs	0.72	0.76	0.80	0.83
*p*,*p*′-DDE	0.46	0.76	0.29	0.28
*cis*-Chlordane	—	—	0.03	−0.02
*trans*-Nonachlor	0.18	0.20	0.11	0.47
Endosulfan	—	—	0.19	−0.20
Heptachlor epoxide	0.72	0.22	0.02	0.10
TriCBs	—	—	0.35	−0.03
TetraCBs	0.51	0.41	0.73	0.37
PentaCBs	0.83	0.89	0.84	0.75
HexaCBs	0.87	0.87	0.82	0.76
HeptaCBs	0.68	0.85	0.72	0.85
OctaCBs	0.32	0.70	0.47	0.81
NonaCBs	—	—	−0.25	—
DecaCBs	—	—	0.40	—
Total PCBs	0.82	0.82	0.81	0.80
Correlation coefficients (*r*) *^b^* between “among tissues” and age vs. UC	0.04	0.75	0.64	—

—, not calculated.

a The values shown were calculated when the detection rate in the tissue was ≥70%.

b Total PCBs were excluded from this calculation.
